# Identifying high risk areas of Zika virus infection by meteorological factors in Colombia

**DOI:** 10.1186/s12879-019-4499-9

**Published:** 2019-10-24

**Authors:** Lung-Chang Chien, Francisco Sy, Adriana Pérez

**Affiliations:** 1 Department of Environmental and Occupational Health, University of Nevada, Las Vegas, School of Public Health, 4505 S. Maryland Parkway, Las Vegas, NV 89154 USA; 2 Department of Biostatistics and Data Science, The University of Texas Health Science Center at Houston-UTHealth, School of Public Health, Austin Campus, 1616 Guadalupe, Suite 6.300, Austin, TX 78071 USA

**Keywords:** Zika virus infection, Meteorological factors, Geographic disparities, Colombia

## Abstract

**Background:**

Several Zika virus (ZIKV) outbreaks have occurred since October 2015. Because there is no effective treatment for ZIKV infection, developing an effective surveillance and warning system is currently a high priority to prevent ZIKV infection. Despite *Aedes* mosquitos having been known to spread ZIKV, the calculation approach is diverse, and only applied to local areas. This study used meteorological measurements to monitor ZIKV infection due to the high correlation between climate change and *Aedes* mosquitos and the convenience to obtain meteorological data from weather monitoring stations.

**Methods:**

This study applied the Bayesian structured additive regression modeling approach to include spatial interactive terms with meteorological factors and a geospatial function in a zero-inflated Poisson model. The study area contained 32 administrative departments in Colombia from October 2015 to December 2017. Weekly ZIKV infection cases and daily meteorological measurements were collected. Mapping techniques were adopted to visualize spatial findings. A series of model selections determined the best combinations of meteorological factors in the same model.

**Results:**

When multiple meteorological factors are considered in the same model, both total rainfall and average temperature can best assess the geographic disparities of ZIKV infection. Meanwhile, a 1-in. increase in rainfall is associated with an increase in the logarithm of relative risk (logRR) of ZIKV infection of at most 1.66 (95% credible interval [CI] = 1.09, 2.15) as well as a 1 °F increase in average temperature is significantly associated with at most 0.79 (95% CI = 0.12, 1.22) increase in the logRR of ZIKV. Moreover, after controlling rainfall and average temperature, an independent geospatial function in the model results in two departments with an excessive ZIKV risk which may be explained by unobserved factors other than total rainfall and average temperature.

**Conclusion:**

Our study found that meteorological factors are significantly associated with ZIKV infection across departments. The study determined both total rainfall and average temperature as the best meteorological factors to identify high risk departments of ZIKV infection. These findings can help governmental agencies monitor at risk areas according to meteorological measurements, and develop preventions in those at risk areas in priority.

## Background

Since October 2015, several outbreaks of Zika virus (ZIKV) have occurred in Central and South America, the Caribbean, Central Africa, and Southeast Asia [1, 2]. The infection of ZIKV may not be lethal, and most infected people may not feel very sick, with only mild symptoms [3]. Compared to the other vector-borne diseases transmitted by *Aedes* mosquitoes, such as dengue fever, yellow fever, or chikungunya, ZIKV infection may not be fatal. The predominant adverse impact of ZIKV infection affects infected pregnant women, who may give birth to infants with microcephaly [4]. Besides mosquitoes, ZIKV can also be transmitted through sexual contact, blood transfusion, and prenatally from mother-to-fetus transmission via pregnancy [5]. Because there is no effective treatment for ZIKV infection, developing an effective surveillance and warning system is currently a high priority to prevent ZIKV infection [6–8].

As a vector-borne disease, ZIKV is strongly associated with increases in the mosquito population. To prevent ZIKV infection, it is essential to break the transmission of the virus from mosquitoes to humans [9]. However, controlling the increase and distribution of vector population is difficult. *Aedes* mosquitoes grow in large numbers when the diurnal temperature is between 30 and 35 °C (i.e., 86-95 °F) [10] and with an optimal temperature to produce the most amount of offspring at 29.2 °C (i.e., 84.56 °F) [11]. Similarly, rainfall may provide a favorable environment to increase the population of *Aedes* mosquitoes [12–14]. Climate change and the increased distribution of *Aedes* mosquitoes are positively correlated [15–17]. With the influence of climate change on diverse areas, the occurrence and distribution of vector-borne diseases, like ZIKV infection, may become more complicated and difficult to monitor.

Spatial analysis and geographic information systems have been used to investigate the distribution of ZIKV infection with related risk factors locally and globally. A study in Brazil, the first country in South America with ZIKV outbreaks, applied a maximum entropy method to analyze the location of observed cases [18]. This literature found that land use was the most important environmental factor to explain the spatial distribution of the risk probability of ZIKV infection. Similar spatial techniques were also applied to other ZIKV prevalent countries, especially in Central and South America. In Puerto Rico, the areas with the highest Zika rates were significantly associated with higher poverty, as identified by the geographically weighted regression [19]. A multi-country study identified nine main municipalities across Colombia, Ecuador, Mexico, and Peru (out of eight countries in Central and South America) as having an increased likelihood of ZIKV transmission by using multiple modeling approaches in ranking the median projected ZIKV infection rate [20]. Some studies applied spatial simulation methods to model potential transmission risk of ZIKV globally, and estimated that tropical and subtropical areas in four continents were experiencing higher risks of ZIKV infection in comparison to non-tropical areas [21, 22].

Since meteorological measurements are known to vary across different areas, there is a need to determine whether climate change will have different impacts on ZIKV infection. Our study aims are to evaluate the spatial association of climate change on ZIKV infection and to identify high-risk areas of ZIKV infection by specific meteorological measurements in Colombia. Our preliminary hypothesis is that the meteorological influence on ZIKV infection significantly varies across different areas. The models were compared to identify the best meteorological factor associated with high-risk areas of ZIKV infection. We also used mapping techniques to present the diverse meteorological influence on ZIKV infection, and to facilitate the inspection of hot zones representing where people were more vulnerable to ZIKV. These maps can clearly identify hot zones with vulnerable populations to ZIKV infection because of the chosen meteorological factor(s). Estimating ZIKV infections when a meteorological factor increased by a particular amount is important for both national and local surveillance systems to assess interventions to decrease the incidence of ZIKV cases.

## Methods

### Data source

We downloaded weekly reports of ZIKV infection cases from the Epidemiological Bulletin, maintained by the Colombian National Institute of Health with a total of 156 consecutive weeks from the 39th week of 2015 (the first week of reporting ZIKV infection) to the 52nd week of 2017. Daily meteorological measurements were fetched from 42 weather monitoring stations in Colombia reported on the Weather Underground (https://www.weatherunderground.com/). The study considered the following meteorological factors: temperature, dew point temperature, relative humidity, sea level pressure, wind speed, and total rainfall. Besides wind speed and total rainfall, each meteorological factor has three types of measurement: maximum, minimum, and average.

### Study area

**Fig. 1:**
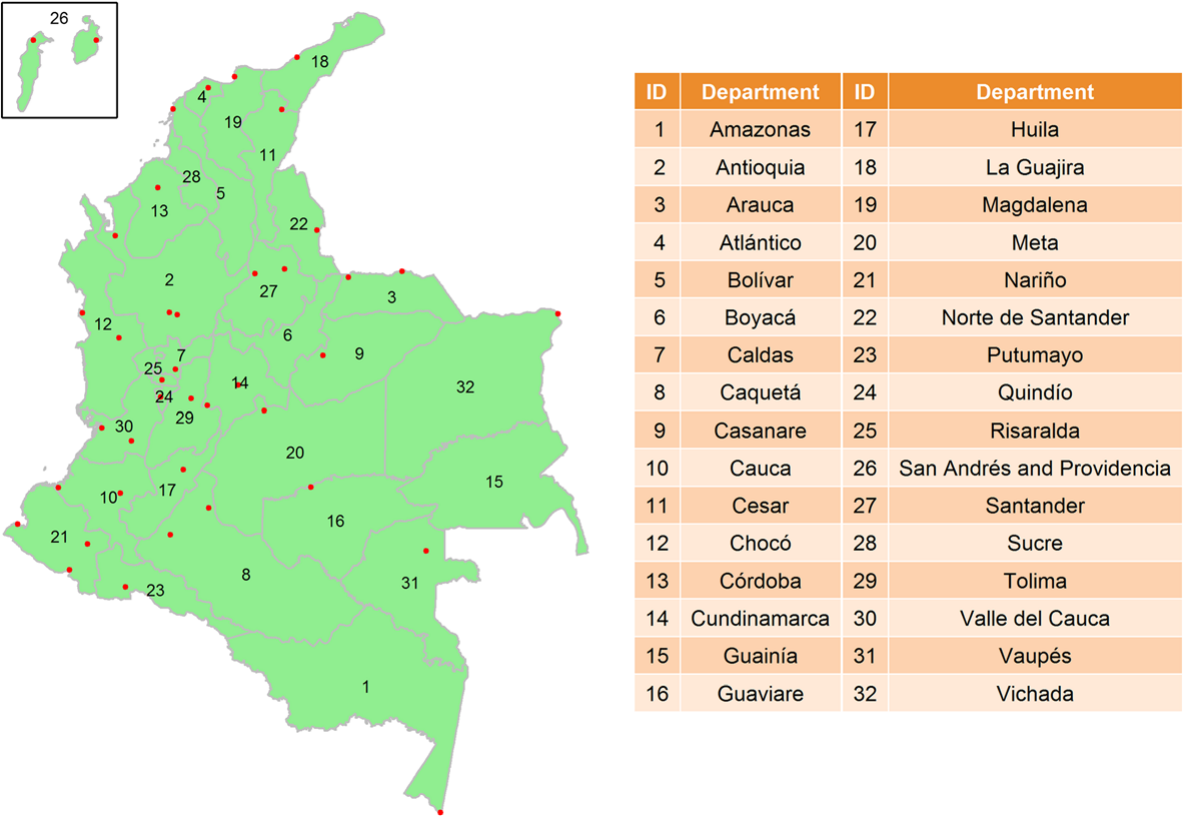
The boundaries of 32 departments and the locations of 42 weather monitoring stations in Colombia

Colombia, the third-most populated country in Latin America, was selected to be the study area because it has very complete records of ZIKV infection cases reported by week and “department” (i.e., the largest administrative area unit and selected as the geographic unit of analysis). Colombia is on the equator, and has a very classical tropical climate with warm and isothermal weather condition. There are a total of 32 departments in Colombia (Fig. 1). Bogotá, the capitol of Colombia, is the most populated area and located within the Cundinamarca’s department with over 10 million residents in 2018 [23], and it is 60% larger than the population in the second populated department. In particular, the archipelago of San Andrés and Providencia is the only department composed of two small islands located in the east of the Caribbean Sea.

### Meteorological measurements

A total of 42 weather monitoring stations are distributed among 32 departments, with 19 departments having multiple stations and 10 departments having only a single station. Only three departments did not have a monitoring station. The locations of all monitoring stations are presented in Fig. 1. In order to unify the time scale between ZIKV infection and meteorological factors as well as dealing with missing data in some of the meteorological factors, a series of data management and imputations were conducted. First, we integrated daily meteorological measurements in the 19 departments with multiple monitoring stations. For instance, daily maximum temperature in a department was determined by the largest value of daily maximum temperatures among all monitoring stations in the same department. Similarly, we adopted the same criterion to determine the daily minimum or average measurement of a meteorological factor by department. For rainfall, we used the maximum value of multiple daily rainfall records from all monitoring stations in a department. Second, we applied a local space-time kriging method to impute missing daily meteorological measurements among 29 departments with at least one monitoring station [24]. Third, we calculated weekly meteorological measurements in each of those 29 departments. Finally, we applied the local space-time kriging method again on the weekly meteorological measurements to impute missing data in the three departments without a monitoring station. The last step made a sample size of 156 weeks× 32 department = 4992 data points for each meteorological factor, which was merged with ZIKV infection data.

### Spatial modeling approach

By defining *Y*_*dt*_ as the number of ZIKV infection cases at time *t* in department *d*, we assumed the distribution of *Y*_*dt*_ as a Poisson distribution with a mean parameter *μ*_*dt*_. Initially, the mean parameter was analyzed using a Poisson model, but because of excessive zero counts in the data of ZIKV infection cases, the zero-inflated Poisson model was chosen. In particular, in order to evaluate the spatial association of meteorological factors on ZIKV infection, we applied a Bayesian structured additive regression modelling approach to incorporate both the zero-inflated Poisson model and the spatial components into account [25]. The mean parameter *μ*_*dt*_ is estimated by the following equation:

$$ \log \left({\mu}_{dt}\right)=\alpha +f(t)+\beta (Year)+(Meteo)\times {f}_{mrf}(d)+{f}_{gsp}\left({lon}_d,{lat}_d\right)+\mathrm{offset},t=1\dots, 156;d=1,\dots, 32 $$

where *α* is the fixed intercept, and the offset is the logarithm of the population of each department. In order to control for temporal autocorrelations, the model contained a time smoother *f*(t) by using a B-spline with the second order random walk prior, and *Year* as a confounding variable with two levels in year 2016 and 2017 (i.e., year 2015 was the reference level). The term *Meteo* was a meteorological random effect interacting with a spatial function *f*_*mrf*_(*d*), which is the Markov random fields (MRF) to conduct a geographic weight in each department. Finally, the model included a geospline function *f*_*gsp*_(*lon*_*d*_, *lat*_*d*_) to evaluate the excessive risk of ZIKV infection which cannot be explained by meteorological factors, where (*lon*_*d*_, *lat*_*d*_) is the centroid coordinate (longitude and latitude) of department *d*.

All unknown parameters in the model were estimated via Markov chain Monte Carlo simulations with 70,000 iterations based on a fully Bayesian inference. The first 20,000 iterations were burned, and we drew estimates from every 50 iterations out of the remaining 50,000 iterations [26]. Therefore, each parameter had 1000 estimates of the posterior distribution, where the posterior mean, the 2.5th and 97.5th percentiles are reported as the estimated parameter and 95% credible interval (CI), respectively. The posterior mean can be explained as the logarithm of relative risk (logRR) for ZIKV infection, and the statistical significance of logRR was determined by whether the 95% CI is strictly different from 0.

The univariate analysis consisted of fitting a model with each meteorological factor to evaluate geographic disparities of ZIKV infection affected by each factor individually. Then, a multivariable model was built to consider at most three meteorological factors simultaneously. The best model was determined by the smallest deviance information criterion (DIC). We did not include highly correlated meteorological factors in the same model to prevent possible multicollinearity within each department. The spatial estimate in *f*_*mrf*_(*d*) represents the expected increase in the logRR of ZIKV infection for a one-unit increase in a meteorological factor in department *d*. The spatial estimate in *f*_*gsp*_(*lon*_*d*_, *lat*_*d*_) can be also regarded as a logRR to quantify the excessive risk of ZIKV infection of a department compare to the average of the whole Colombia after controlling for meteorological factors. The exponentiation of the standard deviation of the spatial estimated conducted by *f*_*mrf*_(*d*) (i.e., $\exp \left(\sqrt{\sigma_{MRF}^2}\right)$) can be explained as the average variation of relative risk due to the meteorological factor among 32 departments. Thus, a geographic disparity percentage (GD%) can be calculated to explain the departmental ZIKV risk on average $\left(\exp \left(\sqrt{\sigma_{MRF}^2}\right)-1\right)\times 100\%$ higher or lower than the overall ZIKV risk. The same way to quantify the level of geographic disparities was also applied in the geospatial function *f*_*gsp*_(*lon*_*d*_, *lat*_*d*_). A larger GD% represents greater geographic disparities among 32 departments.

Data management and summary statistics were implemented in SAS V9.3 (SAS Institute Inc., Cary). Data imputation, model fitting, and mapping were implemented in the R software, version 3.5.0 [27] and BayesX version 2.0 [28].

## Results

**Fig. 2:**
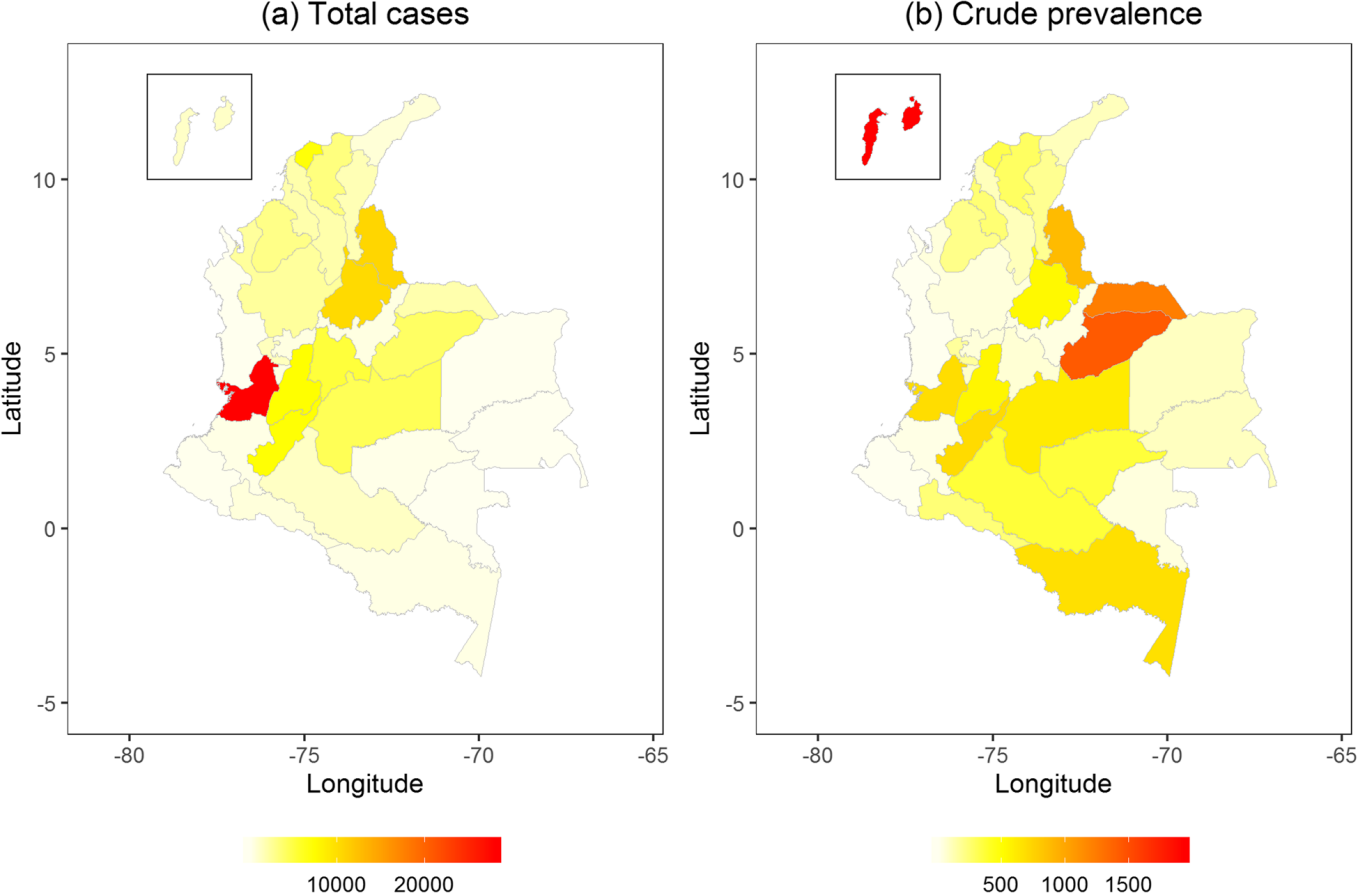
The geographic distribution of (**a**) the total Zika virus infection cases and (**b**) the crude prevalence of Zika virus infection per 10,000 population by each department in Colombia from 2015 to 2017

The summary statistics in Table 1 shows that the weekly average of ZIKV infection cases in Colombia was 21.33 cases (standard deviation = 101.28), and can reach as high as 1750.00 cases, which occurred in Valle del Cauca. Extreme heat and rainfall, and high humidity were also observed from 2015 to 2017. Figure 2a shows that Valle del Cauca, Norte de Santander, and Santander reported over 10,000 cases of ZIKV infection from 2015 to 2017. Figure 2b shows that a high crude incidence of ZIKV infection over 100 cases per 10,000 population appeared in the Islands of San Andrés and Providencia and the departments of Casanare and Arauca. The detailed values of each department in Fig. 2 can be referred to Additional file 1: Table S1. The weekly average of each meteorological measurement by the department is presented in Additional file 1: Fig. S1. The absolute Pearson correlation coefficients of the meteorological factors are greater or equal to 0.7 among all kinds of temperature, dew point temperature, and sea level pressure (see Additional file 1: Table S2).

**Table 1:** Descriptive statistics of weekly Zika virus infection cases and meteorological factors per department

	Mean	SD	Min	Q1	Median	Q3	Max
ZIKV cases	21.33	101.28	0.00	0.00	0.00	3.00	1750.00
Temperature (°F)							
Maximum	89.11	7.07	66.00	86.83	90.00	93.00	137.00
Minimum	65.09	9.17	5.00	61.00	66.36	72.00	81.00
Average	77.60	7.02	53.86	74.74	79.06	82.37	91.43
Dew point temperature (°F)							
Maximum	74.01	6.08	47.00	70.88	74.97	79.00	90.00
Minimum	61.40	10.91	0.00	55.53	64.00	70.00	77.00
Average	69.01	6.95	40.57	65.43	70.16	74.15	80.29
Relative humidity (%)							
Maximum	96.35	5.01	61.00	94.00	100.00	100.00	100.00
Minimum	41.03	11.97	4.00	33.21	42.96	50.00	73.00
Average	73.19	8.94	31.29	68.86	74.57	79.25	93.14
Sea level pressure (Hg)							
Maximum	30.08	0.17	29.79	29.96	30.04	30.15	31.32
Minimum	29.78	0.16	28.73	29.69	29.76	29.85	30.30
Average	29.95	0.14	29.65	29.84	29.92	30.01	30.40
Wind speed (mph)							
Maximum	25.17	31.36	3.47	12.00	15.00	21.00	150.00
Average	4.66	2.84	0.29	2.86	4.17	5.60	20.14
Total rainfall (inches)	0.60	2.11	0.00	0.01	0.16	0.52	39.49

Abbreviation: *SD* Standard deviation, *Q1* The first quartile, *Q3* The third quartile

We first evaluated the need of the independent geospatial function in our models, which results in, whichever meteorological factor in the model, having the geospatial function produced a lower DIC from 82,564.40 to 82,787.98, while the models with the geospatial function had a higher DIC from 85,539.96 to 85,708.96. This result provides the support of appending the geospatial function to consider additional geographic disparities of ZIKV infection, which cannot be fully explained by the meteorological factors.

Table 2 shows the proportion of departments in terms of positive, negative and non-significant associations of the MRF univariately for each meteorological factor. A larger proportion of departments had a significant positive association between ZIKV infections and each one of the meteorological factors. For example, the average sea level pressure and the total rainfall were associated with increasing ZIKV infections on 17 departments (53.13%). The largest meteorological impact on ZIKV infection was observed in Amazonas, where the expected increase in logRR for 1-mph increase in the maximum wind speed was 2.28 (95% CI = 0.24, 2.98). From the perspective of model evaluation, both the average temperature and average dew point temperature better assessed the geographic disparities of ZIKV infection because both models have the lowest DIC by 82,560.97 and 82,558.50, respectively.

**Table 2:** The summary of spatial estimates from the Markov random fields in terms of significant positive (the logarithm of relative risk (logRR) is significantly greater than 0), significant negative (logRR is significantly smaller than 0) and non-significane (logRR is not different from 0) and geographic disparity percentage in each meteorological factor based on the univariate analysis

	Significant positive			Significant negative		Non-significance		
	N	n (%)	logRR range	n (%)	logRR range	n (%)	logRR range	GD%
Temperature								
Maximum	32	12 (37.50)	(0.29, 2.00)	7 (21.88)	(−4.00, −0.38)	13 (40.63)	(−0.53, 1.01)	252.27
Minimum	32	16 (50.00)	(0.51, 1.73)	9 (28.13)	(− 4.61, − 0.47)	7 (21.88)	(− 0.83, 0.42)	303.00
Average	32	16 (50.00)	(0.42, 1.98)	9 (28.13)	(−4.85, −0.44)	7 (21.88)	(−0.74, 1.36)	300.33
Dew point temperature								
Maximum	32	15 (46.88)	(0.34, 1.84)	7 (21.88)	(−4.65, −0.30)	10 (31.25)	(−0.93, 0.58)	305.88
Minimum	32	15 (46.88)	(0.46, 1.77)	7 (21.88)	(−3.87, −0.20)	10 (31.25)	(−1.14, 0.26)	276.23
Average	32	14 (43.75)	(0.73, 2.11)	7 (21.88)	(−4.55, −1.01)	11 (34.38)	(−1.12, 0.68)	328.94
Relative humidity								
Maximum	32	14 (43.75)	(0.57, 1.86)	8 (25.00)	(−3.94, −0.41)	10 (31.25)	(−1.00, 0.87)	272.83
Minimum	32	13 (40.63)	(0.72. 1.41)	6 (18.75)	(−3.97, −0.60)	13 (40.63)	(−1.09, 1.41)	291.87
Average	32	15 (46.88)	(0.52. 1.87)	8 (25.00)	(−4.48, −0.29)	9 (28.13)	(−0.46, 0.43)	256.35
Sea level pressure								
Maximum	32	9 (28.13)	(0.44, 2.09)	7 (21.88)	(−4.59, −0.69)	16 (50.00)	(−0.20, 1.85)	299.55
Minimum	32	14 (43.75)	(0.46, 1.85)	8 (25.00)	(−4.27, −0.46)	10 (31.25)	(−0.62, 1.85)	328.66
Average	32	17 (53.13)	(0.46, 1.75)	8 (25.00)	(−5.32, −0.24)	7 (21.88)	(−0.24, 0.37)	277.08
Wind speed								
Maximum	32	15 (46.88)	(0.25, 2.28)	6 (18.75)	(−4.88, −0.53)	11 (34.38)	(−1.22, 0.73)	327.43
Average	32	14 (43.75)	(0.40, 1.71)	9 (28.13)	(−4.71, −0.55)	9 (28.13)	(−0.34, 0.61)	296.03
Rainfall	32	17 (53.13)	(0.45, 1.65)	7 (21.88)	(−4.74, −0.23)	8 (25.00)	(−0.87, 0.20)	282.23

Abbreviation: *logRR* The logarithm of relative risk, *GD%* Geographic disparity percentage

In the univariate analysis, geographic disparities of ZIKV infection due to meteorological factors measured by GD% diversely ranged from 252.27% due to maximum temperature to 328.93% due to average dew point temperature. Maps in Additional file 1: Figure S2 reveal that the meteorological influence on ZIKV infection had different levels of geographic disparities across 32 departments in Colombia. According to the corresponding significance maps shown in Additional file 1: Figure S3, most at-risk departments due to meteorological factors were located in the northern and eastern areas of Colombia. In addition, the southern areas of Colombia may be also at risk because of the minimum temperature, average relative humidity, maximum wind speed, and total rainfall. Four departments (Norte de Santander, Risaralda, Tolima, and Valle del Cauca) located in western Colombia were detected as at-risk areas by all 15 meteorological factors. On the contrary, 10 departments were never detected as at-risk areas by any of those meteorological factors, and most of them are located along the coast line of the Pacific Ocean and along the border of Brazil and Venezuela.

**Fig. 3:**
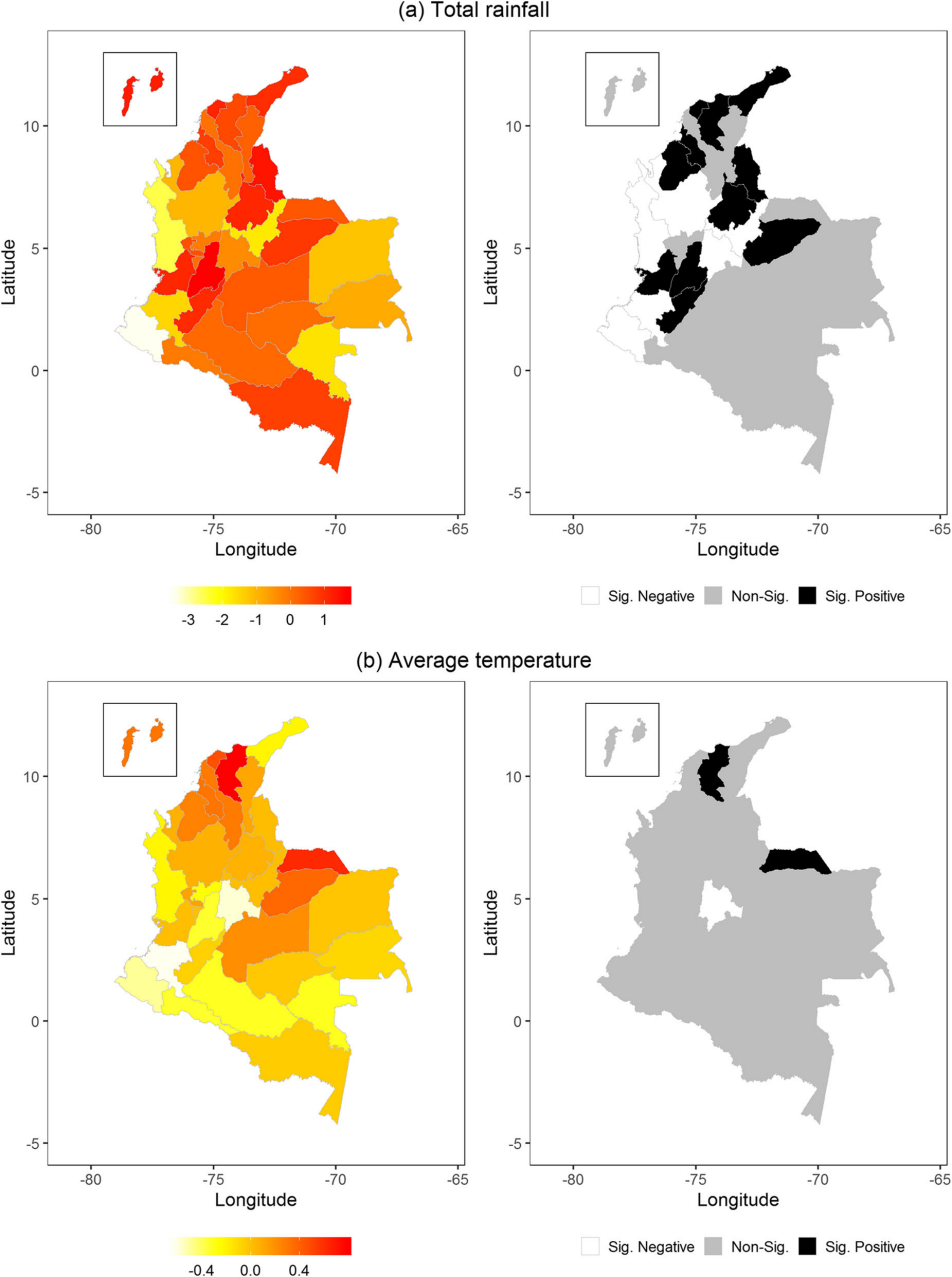
The spatial influence of (**a**) total rainfall and (**b**) average temperature on Zika virus infection at the department level in Colombia. The left maps are the logarithm of relative risk (logRR) estimated by the Markov random fields in the multivariate analysis, and the right maps are the significance of logRR

**Fig. 4:**
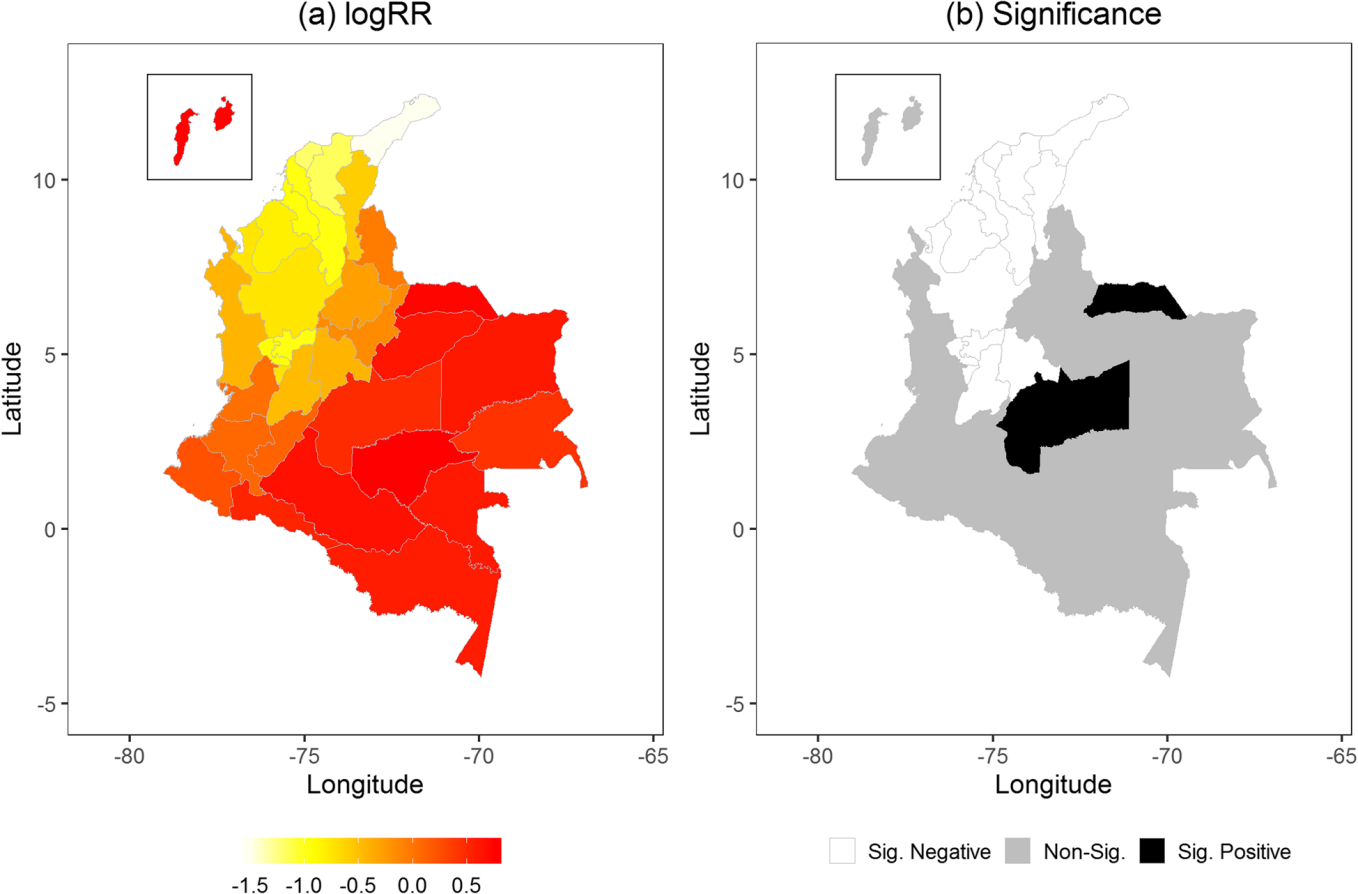
The spatial distribution of (**a**) the logarithm of relative risk (logRR) estimated from the geospline function and (**b**) the significance of logRR. Both are based on the multivariate analysis

The multivariable analysis shows that both total rainfall and average temperature in the same model had the lowest DIC by 82,528, which is much farther below the DICs of the two best models in the univariate analysis. Figure 3a shows that the association of total rainfall and ZIKV infection in the northern areas of Colombia. Twenty of 32 departments (62.5%) had a positive association between total rainfall and ZIKV infection, while only 11 of them were statistically significant. Most of these 11 departments are located in northern Colombia, while Tolima located in central Colombia had the largest impact (logRR = 1.66; 95% CI = 1.09, 2.15). Figure 3b presents a positive association between average temperature and ZIKV infection in northern and northeastern Colombia, while the significance map only reveals two departments having a significant positive impact on ZIKV: Magdalena (logRR = 0.79; 95% CI = 0.12, 1.22) and Arauca (logRR = 0.62; 95% CI = 0.28, 1.04). After controlling for both total rainfall and average temperature, 15 departments had a positive logRR calculated from the geospatial function, revealing that those departments had excessive ZIKV risks, see Fig. 4a; however, Fig. 4b shows that only Arauca and Meta had a logRR significantly larger than 1. Comparing the two selected meteorological factors, the total rainfall resulted in a higher GD% than average temperature (305.69% vs. 10.99%).

## Discussion

As the alarm of ZIKV has arose since 2015, the research on ZIKV vaccines and antiviral drugs is still being conducted, despite its severity has been gradually diminishing since 2017 [29]. Some preventive interventions have been implemented in different areas using the same vector control strategies applied to dengue fever, which is also transmitted by *Aedes* mosquitoes [30]. Those strategies may not prevent the occurrence of outbreaks and cannot predict when an outbreak will occur. A systematic review in 2016 has pointed out the need to define the range of ZIKV vectors and to identify new areas where ZIKV transmission could take place to enable early transmission [31]. Our study provides a statistical approach to monitor climate change for quantifying the impact of ZIKV infection in a large area by meteorological measurements, which are collected in multiple weather stations routinely. Our main findings are: First, different meteorological factors can detect different at-risk areas, and total rainfall and average sea level pressure can aggressively detect more at-risk areas; second, when considering multiple meteorological factors in the same model, using both total rainfall and average temperature, can better detect at-risk areas; third, meteorological factors may not totally explain the variation of ZIKV infection, and there might be some other unobserved risk factors which may contribute to excessive ZIKV risk infection.

Temperature and rainfall have been linked to *Aedes albopictus* or *Aedes aegypti* population in literature [32, 33], while the direct evidence is still limited to link the association between ZIKV infection and the two meteorological factors. Our findings are consistent with the literature in vector-borne diseases and the population of *Aedes albopictus* or *Aedes aegypti*. We did not exclude the possibility that other meteorological measurements were associated with vector-borne diseases [34, 35]. This study proposed a systematic procedure to filter the best meteorological factors; however, for a more comprehensive and aggressive prevention on ZIKV, using the univariate model is still acceptable. In confronting emerging ZIKV outbreaks with limited time and resources available, public health officials will have to rely on the best meteorological factors as an option to determine where to focus their preventive interventions.

The literature has shown the geographic variations of ZIKV infection. For instance, a study analyzed individual ZIKV cases in Nicaragua, revealing a 10–15% difference in the risk of ZIKV infections across neighborhoods [36]. However, the study area in this research is relatively small at around 3 km^2^. A global health study applied the niche modelling techniques to estimate the potential geographic distribution area of *Aedes aegypti*, resulting in a high ZIKV risk concentration in the countries surrounding the Caribbean Sea and in the Atlantic coast of South America [21]. Based on their modeling estimations, Colombia may have at least 10 million populations potentially exposed to ZIKV. The spatial variation of ZIKV in Colombia indeed has been verified in the literature [37–39], while no direct evidence shows that the spatial variation of ZIKV was associated with climate change. Our findings on the best meteorological predictors are consistent with the results of a previous study, which analyzed the data in the whole Latin America [40]. In this study, the meteorological predictors can only explain 36-39% of the spatial variation of ZIKV, revealing that there are still additional non-meteorological factors which can explain the spatial variation of ZIKV. This scenario emphasizes the importance of having a specific function to quantify the spatial variation that is not explained by meteorological measurements, like what the geospatial function can contribute in our models.

The risk factors responsible for emergence and the continued occurrence of ZIKV infection are not limited to meteorological factors. Biomedical research has proven that ZIKV can also be transmitted sexually [41–43]. Environmental and social changes, such as land use and human movement like tourism and immigration, might also be potential risk factors for ZIKV epidemics [43]. These potential risk factors are difficult to obtain and aggregate by location and time in a spatiotemporal analysis. Ignoring these unobserved risk factors might hide their influence on ZIKV infection in some areas. Thus, our final model included a geospatial function to catch the proportion of ZIKV incidence unexplained by meteorological factors, resulting in two departments (Arauca and Meta) with excessive risk of ZIKV after controlling for the average temperature and total rainfall. In particular, average temperature was significantly associated with ZIKV in Arauca in Fig. 3, but both meteorological factors were not significantly associated with ZIKV in Meta. This finding indicates the importance of having an additional geospatial function to detect at-risk areas which cannot be detected by meteorological factors. In fact, previous studies have explored non-meteorological risk factors of ZIKV. A risk assessment study of ZIKV applied a hierarchical approach to evaluate climate and nature factors as well as non-meteorological risk dimensions of ZIKV, resulting in that mosquito densities and epidemics of dengue fever are more related to ZIKV transmission than climate and nature factors [44]. Sexual contact has been also verified to be a plausible transmission pathway by case reports [45, 46], despite the fact that results from other laboratory experiments and simulation research are inconsistent [47–49]. To concern the difficulties of collecting non-meteorological data as confounding factors matching the daily ZIKV cases and meteorological measurements by date and department, adding an independent geospatial function in the model provides the rationale of better taking those unobserved risk factors into account.

This study has three main limitations. First, we applied the DIC to select average temperature and total rainfall as the best meteorological predictors to explain the geographic disparities of ZIKV infection, while no R-squared measurement can be calculated to exactly know the explained percentage based on the methodology of the Bayesian model. Second, current ZIKV data were only released based on department, which is not the smallest administrative area in Colombia. Meanwhile, the study is unable to evaluate further risk patterns inside those high risk departments identified by our models. Third, we do not know if the excessive ZIKV cases were imported, so further research is needed because the current database does not distinguish domestic and imported ZIKV cases.

## Conclusions

Although ZIKV infection has gradually decreased since 2017, there is a threat of the resurgence of ZIKV because no effective vaccine or antiviral drugs have been developed yet. The best strategy to confront ZIKV is to implement preventive interventions. The findings of this study can be used by governmental agencies to devote their prevention efforts in identified high risk areas where meteorological factors can predict the increase of ZIKV infections. By combining meteorological factors with a real-time weather monitoring system, future work is to predict possible outbreaks of ZIKV earlier, and to provide timely surveillance and assistance in areas with vulnerable populations.

## Supplementary information

**Additional file 1.** The supplementary materials with a correlation matrix among meteorological measurements, the geographic distribution maps of the averages of meteorological measurement, and the spatial estimates with corresponding significance from the Markov random fields in the univariate analysis.

## Data Availability

Please contact the corresponding author for data requests.

## Abbreviations

CI: Credible interval
DIC: Deviance information criterion
GD%: Geographic disparity percentage
logRR: Logarithm of relative risk
MRF: Markov random fields
ZIKV: Zika virus
