# Supplementary material for: Identifying high risk areas of Zika virus infection by meteorological factors in Colombia
Source: BMC Infect Dis. 2019 Oct 24;19:888. doi: 10.1186/s12879-019-4499-9 (PMC6814059; doi:10.1186/s12879-019-4499-9)

Additional file 1

**Identifying High Risk Areas of Zika Virus Infection by Meteorological Factors in Colombia**

Lung-Chang Chien, Adriana Perez, Francisco S. Sy

Table S1. The summary of ZIKV cases in 32 departments of Colombia, 2015-2017

| **Department** | **Population** | **Total cases** | **Crude incidence^a^** | **Maximum weekly cases (Year-Week)** | **Maximum weekly crude prevalence^a^** |
| --- | --- | --- | --- | --- | --- |
| Amazonas | 46,950 | 316 | 673.06 | 39 (2016**-**09) | 83.07 |
| Antioquia | 5,601,507 | 2,356 | 42.06 | 244 (2016**-**06) | 4.36 |
| Arauca | 153,028 | 1,868 | 1,220.69 | 145 (2016-12) | 94.75 |
| Atlantico | 2,112,001 | 6,802 | 322.06 | 1,265 (2015-52) | 59.90 |
| Bolivar | 1,836,640 | 1,937 | 105.46 | 220 (2015-45) | 11.98 |
| Boyaca | 1,210,982 | 390 | 32.21 | 36 (2016-13) | 2.97 |
| Caldas | 898,490 | 414 | 46.08 | 51 (2016-50) | 5.68 |
| Caqueta | 337,932 | 1,226 | 362.79 | 130 (2016-05) | 38.47 |
| Casanare | 281,294 | 4,013 | 1,426.62 | 396 (2016-18) | 140.78 |
| Cauca | 1,182,022 | 293 | 24.79 | 40 (2016-50) | 3.38 |
| Cesar | 878,437 | 1,642 | 186.92 | 177 (2016-04) | 20.15 |
| Choco | 388,476 | 24 | 6.18 | 8 (2016-13) | 2.06 |
| Cordoba | 1,462,909 | 2,967 | 202.82 | 403 (2016-06) | 27.55 |
| Cundinamarca | 9,007,373 | 5,155 | 57.23 | 594 (2016-08) | 6.60 |
| Guainia | 18,797 | 18 | 95.76 | 12 (2016-21) | 63.84 |
| Guaviare | 56,758 | 203 | 357.66 | 31 (2016-13) | 54.62 |
| Huila | 1,001,476 | 7,022 | 701.17 | 803 (2016-05) | 80.18 |
| La Guajira | 655,943 | 684 | 104.28 | 104 (2016-18) | 15.86 |
| Magdalena | 1,136,819 | 3,206 | 282.01 | 468 (2016-08) | 41.17 |
| Meta | 713,772 | 4,375 | 612.94 | 322 (2016-18) | 45.11 |
| Narino | 1,498,234 | 68 | 4.54 | 8 (2016-10) | 0.53 |
| Norte de Santander | 1,208,336 | 10,588 | 876.25 | 1,167 (2016-03) | 96.58 |
| Putumayo | 237,197 | 573 | 241.57 | 59 (2016-08) | 24.87 |
| Quindío | 518,691 | 403 | 77.70 | 61 (2016-19) | 11.76 |
| Risaralda | 859,666 | 1,428 | 166.11 | 79 (2016-05) | 9.19 |
| San Andres and Providencia | 59,573 | 1,147 | 1,925.37 | 186 (2015-49) | 312.22 |
| Santander | 1,913,444 | 10,253 | 535.84 | 732 (2016-14) | 38.26 |
| Sucre | 762,263 | 1,887 | 247.55 | 220 (2016-16) | 28.86 |
| Tolima | 1,312,304 | 7,199 | 548.58 | 744 (2016-07) | 56.69 |
| Valle del Cauca | 4,052,535 | 27,964 | 690.04 | 1,750 (2016-19) | 43.18 |
| Vaupes | 19,943 | 8 | 40.11 | 3 (2017-36) | 15.04 |
| Vichada | 44,592 | 42 | 94.19 | 5 (2016-19) | 11.21 |

^a^ Per 100,000 population

Table S2. Pearson correlation coefficients among meteorological factors in Colombia

|  | TP_max_ | TP_min_ | TP_avg_ | DP_max_ | DP_min_ | DP_avg_ | RH_max_ | RH_min_ | RH_avg_ | SP_max_ | SP_min_ | SP_avg_ | WS_max_ | WS_avg_ | RF |
| --- | --- | --- | --- | --- | --- | --- | --- | --- | --- | --- | --- | --- | --- | --- | --- |
| TP_max_ | 1.00 | 0.70 | 0.86 | 0.74 | 0.43 | 0.70 | -0.06 | -0.19 | -0.26 | -0.61 | -0.71 | -0.80 | 0.07 | 0.21 | 0.04 |
| TP_min_ |  | 1.00 | 0.90 | 0.68 | 0.65 | 0.83 | -0.16 | 0.20 | -0.05 | -0.81 | -0.57 | -0.85 | 0.01 | 0.19 | -0.01 |
| TP_avg_ |  |  | 1.00 | 0.76 | 0.63 | 0.88 | -0.20 | 0.08 | -0.18 | -0.83 | -0.69 | -0.94 | 0.02 | 0.22 | 0.01 |
| DP_max_ |  |  |  | 1.00 | 0.51 | 0.84 | 0.18 | 0.19 | 0.20 | -0.60 | -0.70 | -0.77 | 0.08 | 0.07 | 0.11 |
| DP_min_ |  |  |  |  | 1.00 | 0.72 | -0.05 | 0.60 | 0.18 | -0.66 | -0.39 | -0.64 | -0.06 | -0.07 | 0.01 |
| DP_avg_ |  |  |  |  |  | 1.00 | 0.06 | 0.39 | 0.27 | -0.79 | -0.64 | -0.89 | 0.01 | -0.01 | 0.07 |
| RH_max_ |  |  |  |  |  |  | 1.00 | 0.14 | 0.59 | 0.16 | -0.01 | 0.09 | 0.10 | -0.21 | 0.14 |
| RH_min_ |  |  |  |  |  |  |  | 1.00 | 0.64 | -0.22 | 0.02 | -0.14 | -0.09 | -0.28 | 0.06 |
| RH_avg_ |  |  |  |  |  |  |  |  | 1.00 | 0.04 | 0.07 | 0.05 | 0.02 | -0.38 | 0.15 |
| SP_max_ |  |  |  |  |  |  |  |  |  | 1.00 | 0.57 | 0.87 | 0.02 | -0.11 | 0.01 |
| SP_min_ |  |  |  |  |  |  |  |  |  |  | 1.00 | 0.76 | -0.05 | -0.03 | -0.05 |
| SP_avg_ |  |  |  |  |  |  |  |  |  |  |  | 1.00 | -0.02 | -0.11 | -0.04 |
| WS_max_ |  |  |  |  |  |  |  |  |  |  |  |  | 1.00 | 0.24 | 0.05 |
| WS_avg_ |  |  |  |  |  |  |  |  |  |  |  |  |  | 1.00 | -0.07 |
| RF |  |  |  |  |  |  |  |  |  |  |  |  |  |  | 1.00 |

Abbreviation: TP_max_ = Maximum temperature; TP_min_ = Minimum temperature; TP_avg_ = Average temperature; DP_max_ = Maximum dew point temperature; DP_min_ = Minimum dew point temperature; DP_avg_ = Average dew point temperature; RH_max_ = Maximum relative humidity; RH_min_ = Minimum relative humidity; RH_avg_ = Average relative humidity; SP_max_ = Maximum sea level pressure; SP_min_ = Minimum sea level pressure; SP_avg_ = Average sea level pressure; WS_max_ = Maximum wind speed; WS_avg_ = Average wind speed; RF = Total rainfall

Figure S1. Maps of the average of each meteorological measure by each department in Colombia.


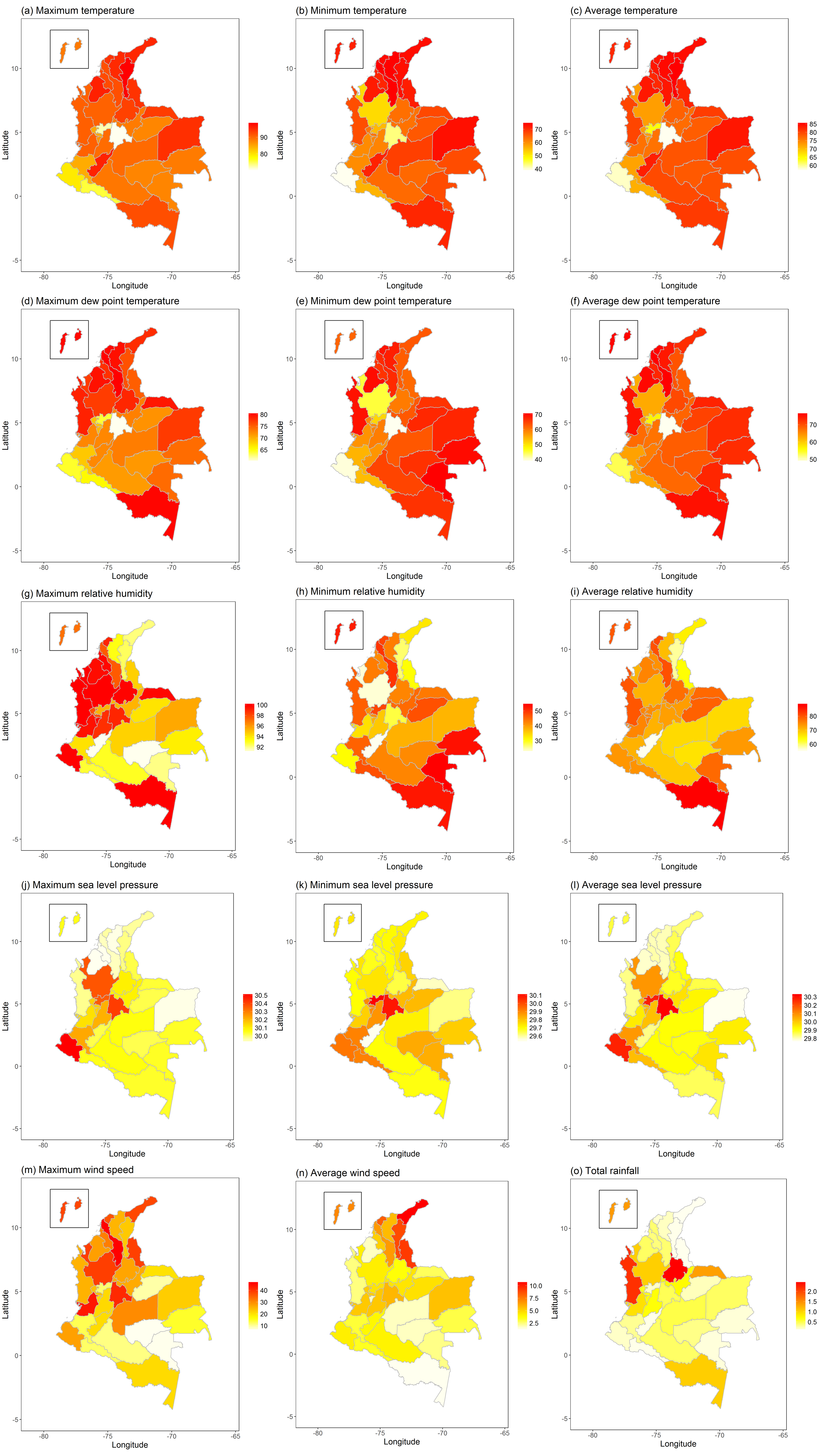


Figure S2. The spatial influence of meteorological measurements on Zika virus infection at the department level in Colombia. The spatial estimates were conducted by the Markov random fields in the univariate analysis.


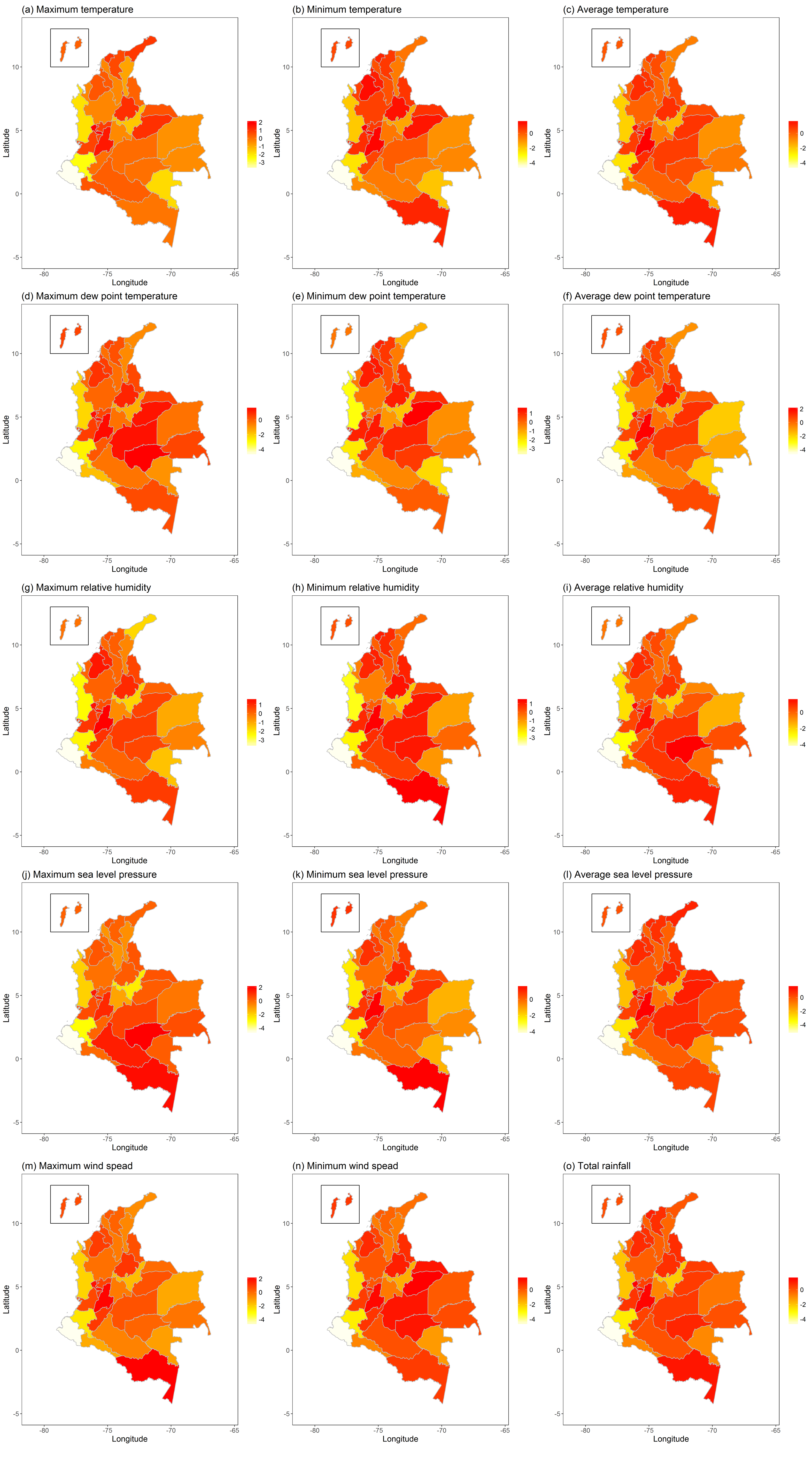


Figure S3. The significance of spatial estimates from the Markov random fields in the univariate analysis.


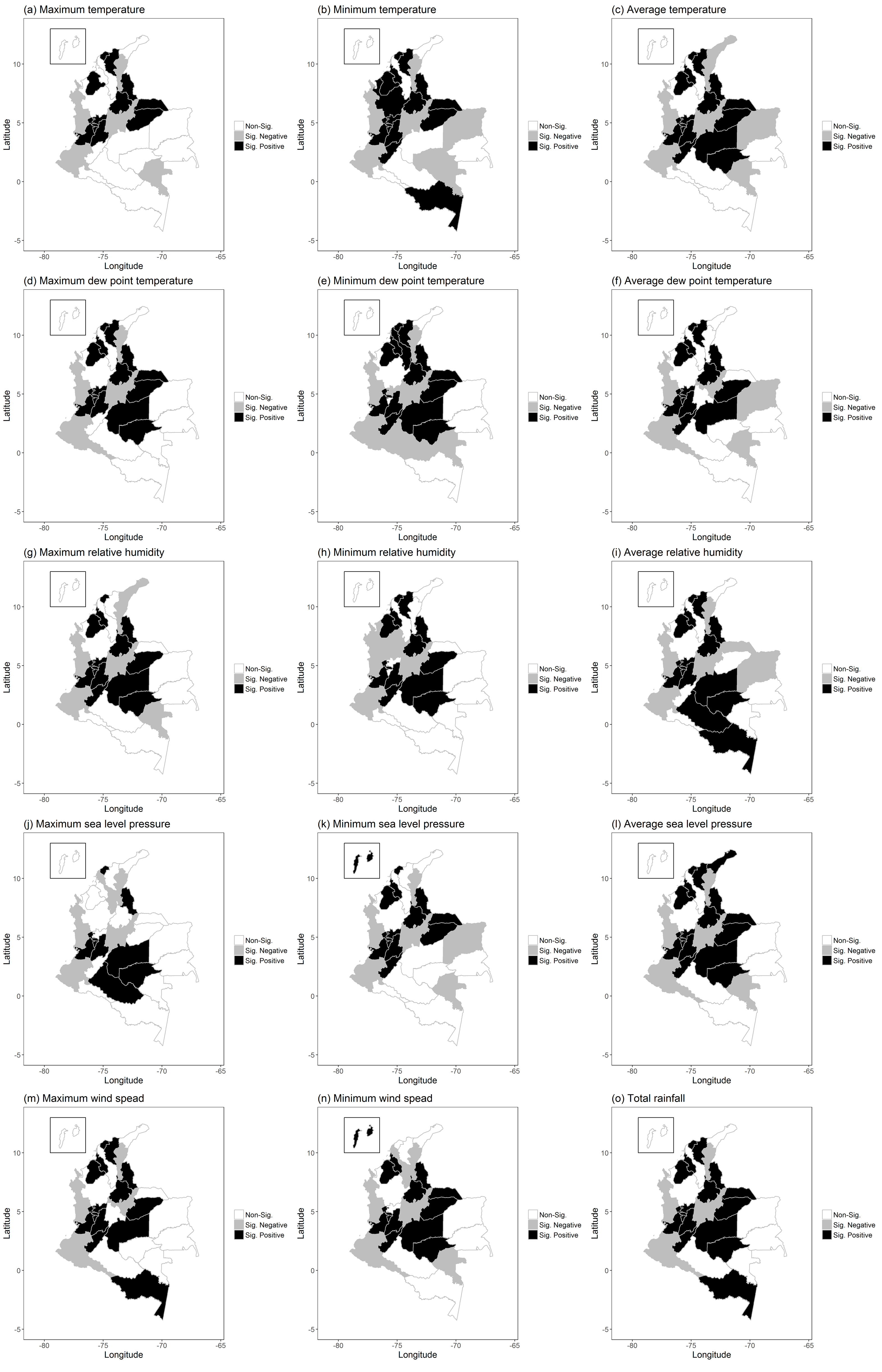

Supplement: Supplementary file 1 — Additional file 1. The supplementary materials with a correlation matrix among meteorological measurements, the geographic distribution maps of the averages of meteorological measurement, and the spatial estimates with corresponding significance from the Markov random fields in the univariate analysis. [file 12879_2019_4499_MOESM1_ESM.docx]
